# Quantifying intracellular glucose levels when yeast is grown in glucose media

**DOI:** 10.1038/s41598-023-43602-z

**Published:** 2023-10-10

**Authors:** Xiang Li, Matthias Heinemann

**Affiliations:** 1 Molecular Systems Biology, Groningen Biomolecular Sciences and Biotechnology Institute, University of Groningen, 9747 AG Groningen, The Netherlands; 2 Present Address: Department of Life Science, Division of Systems and Synthetic Biology, Chalmers University of Technology, Kemigården 1, SE-412 96 Göteborg, Sweden

**Keywords:** Metabolomics, Microbiology techniques, Industrial microbiology

## Abstract

In *Saccharomyces cerevisiae*, intracellular glucose levels impact glucose transport and regulate carbon metabolism via various glucose sensors. To investigate mechanisms of glucose sensing, it is essential to know the intracellular glucose concentrations. Measuring intracellular glucose concentrations, however, is challenging when cells are grown on glucose, as glucose in the water phase around cells or stuck to the cell surface can be carried over during cell sampling and in the following attributed to intracellular glucose, resulting in an overestimation of intracellular glucose concentrations. Using lactose as a carryover marker in the growth medium, we found that glucose carryover originates from both the water phase and from sticking to the cell surface. Using a hexokinase null strain to estimate the glucose carryover from the cell surface, we found that glucose stuck on the cell surface only contributes a minor fraction of the carryover. To correct the glucose carryover, we revisited l-glucose as a carryover marker. Here, we found that l-glucose slowly enters cells. Thus, we added l-glucose to yeast cultures growing on uniformly ^13^C-labeled d-glucose only shortly before sampling. Using GC–MS to distinguish between the two differently labeled sugars and subtracting the carryover effect, we determined the intracellular glucose concentrations among two yeast strains with distinct kinetics of glucose transport to be at 0.89 mM in the wild-type strain and around 0.24 mM in a mutant with compromised glucose uptake. Together, our study provides insight into the origin of the glucose carryover effect and suggests that l-glucose added to the culture shortly before sampling is a possible method that yet has limitations with regard to measurement accuracy.

## Introduction

In *Saccharomyces cerevisiae*, glucose not only fuels the growth of cells but also acts as a signaling molecule, and intracellular glucose could directly impact glucose transport^1^ or indirectly trigger the downstream transcriptional response of glucose metabolism^2^. Since *S. cerevisiae* mainly relies on facilitated diffusion to transport glucose, the accumulation of intracellular glucose can increase glucose efflux and, therefore, can reduce the net glucose influx^3,4^, which occurs in derepressed yeast cells^1^. On the other hand, glucose is also known to be an intracellular signal triggering downstream transcriptional responses with various intracellular glucose sensors perceiving intracellular glucose levels and activating signal transduction pathways to rewire expression levels^2,5,6^. Finally, it has also been reported that intracellular accumulation of glucose is adverse to cell growth, likely due to osmotic pressure effects^7,8^. Thus, overall, to better understand the physiological roles of intracellular glucose in glucose transport and metabolism, it is critical to reliably determine intracellular glucose levels.

**Figure 1:**
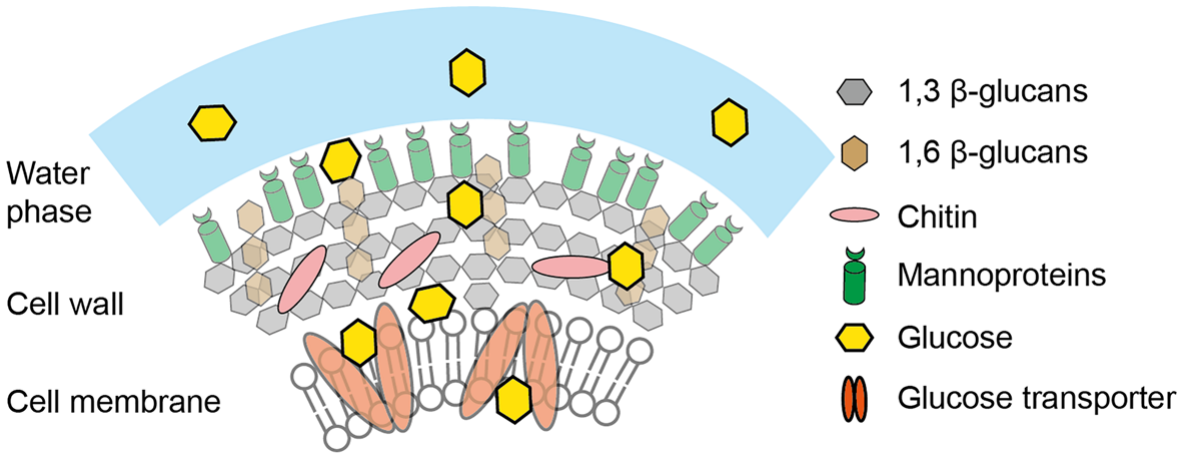
Scenarios where extracellular glucose could be situated and carried over. The path of glucose transport and two possible scenarios from which the carried-over glucose could originate. Scenario 1: in glucose-containing media, glucose could be situated in the water phase between cells in the cell pellets. Scenario 2: glucose could attach to the cell wall, cell membrane, or glucose transporters.

However, despite the importance of intracellular glucose levels, measuring intracellular glucose concentrations is still challenging in cases when glucose is present in the medium as a carbon source. This is because the measurements of intracellular glucose concentrations are confounded by extracellular glucose carried over from the culture medium during the processing of cells for intracellular metabolite analysis^1^. Extracellular glucose could be carried over in either the water phase surrounding pelleted cells (Fig. 1, scenario 1) or via binding of glucose to the cell wall, cell membrane, or glucose transporters (Fig. 1, scenario 2), or via a combination of both. Extensive washing of cells with a culture medium without glucose cannot be applied, as in this case, the intracellular metabolite levels will change due to continued metabolic activity. Alternatively, extensive washing of the quenched cell pellet with quenching solutions, such as organic solvents at very low temperatures, may lead to the leakage of metabolites from yeast cells^9^. In batch cultures, the carryover problem is further aggravated by the fact that the extracellular glucose levels are much higher than the physiological intracellular glucose levels, meaning that even a minor carryover of extracellular glucose will heavily distort the measurements.

In principle, genetically encoded molecular glucose sensors, e.g., based on Förster resonance energy transfer, could be used to determine the cytosolic glucose in yeast cells^10^. However, the concentration range that such sensors can detect is usually limited, and estimating the absolute levels requires assumptions and calibration^11,12^. For instance, to determine absolute intracellular glucose concentrations, an approach based on enzymatic assays or metabolomics is still required, which then requires addressing the challenge of glucose carryover. In turn, solving the carryover issue is difficult as we do not know the precise cause of the carryover: Does it mainly originate from the water phase or from glucose sticking to the cell wall or membrane? Researchers have used l-glucose as a marker for carryover, assuming that it is not being taken up by the budding yeast^1^. However, others have found that l-glucose is taken up^13,14^, making using l-glucose as a marker questionable. Thus, is there any alternative to l-glucose as the marker? Furthermore, when should the carryover marker be added to the medium, and how will this influence the overall results?

Here, to investigate the source of glucose carryover, we first used lactose as a marker in the medium. We found that glucose carryover originates from both the water phase and from sticking to the cell surface. To further understand the contributions of the glucose stuck on the cell surface, we then constructed a hexokinase null strain and estimated glucose carryover from the sticking effect, finding that glucose on the cell surface only contributes a minor fraction of the total carryover. Eventually, we revisited l-glucose as a glucose carryover marker and determined the dynamics of l-glucose uptake. We found that l-glucose was a suitable marker for correction of the carryover if it is added to the steady-state culture shortly before quenching, providing a practical way of measuring intracellular glucose concentrations while correcting the carryover effect.

## Results

### A carryover marker is essential when determining intracellular glucose concentration

**Figure 2:**
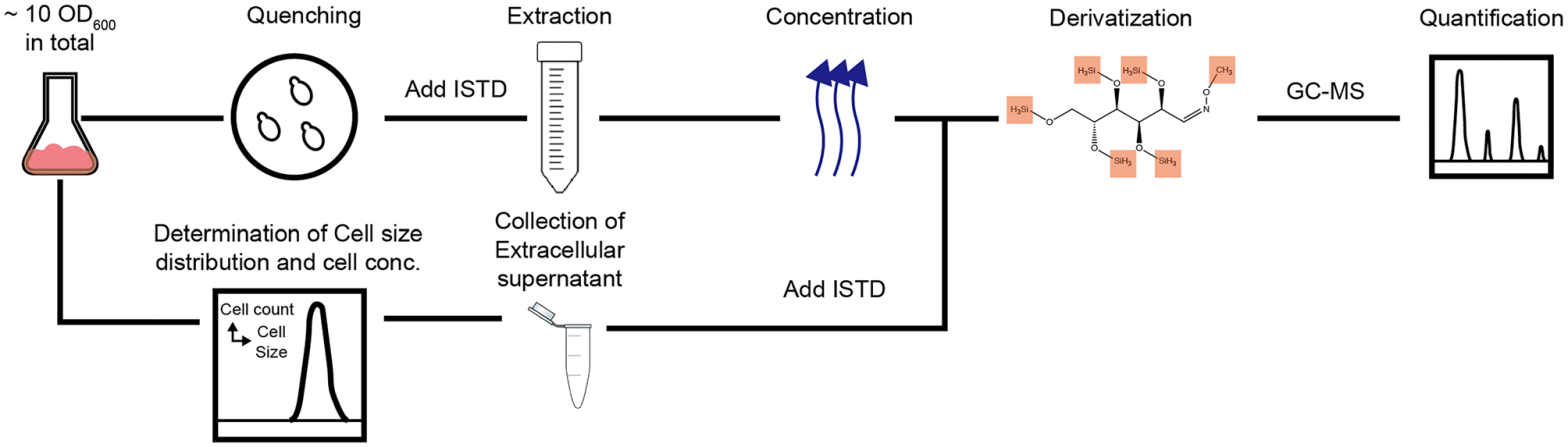
The workflow of the sampling-quantification procedures. Cell samples were quenched with cold methanol and extracted with boiling ethanol, and cell extract was concentrated and derivatized before being quantified with GC–MS. By the time of sampling for intracellular metabolites, cell counts and cell size distributions were determined by a cell counter and analyzer (CASY). At the same time, the supernatant of the culture was also collected and analyzed in the same way as the intracellular samples. ISTD: Internal Standard; GC–MS: Gas Chromatography-Mass Spectrometry.

First, we reassessed the necessity of a carryover marker for determining intracellular glucose concentrations when budding yeast is grown on glucose as a carbon and energy source. To this end, we took a sample from an exponentially growing culture, quenched this sample with cold methanol, and extracted the metabolites using boiling ethanol. After the extraction, the intracellular metabolites were derivatized, and GC–MS was used to quantify concentrations of metabolites. At the time of sampling, the concentrations of extracellular metabolites were also determined, as well as the cell density and size distributions in the culture (Fig. 2).

Here, in line with previous findings^15^, we found that with this approach, the intracellular glucose concentration would surpass the extracellular glucose concentrations (Fig. S1). We considered this not possible as *S. cerevisiae* relies on facilitated diffusion to transport glucose^4,16,17^, for which a concentration gradient from outside (high) to inside (low) is needed. This result shows that extracellular glucose must be carried over, meaning that correction of carryover is required to obtain correct intracellular glucose concentrations.

An extracellular marker compound added to the medium could be used to correct the glucose that is carried over from the extracellular environment. A suitable carryover marker compound should fulfill the following criteria: (1) the marker should not be produced by the cell itself; (2) it should not enter cells or at least much slower than the substrate glucose; (3) the marker should have the same stickiness to the cell wall or cell membrane as glucose; (4) if mass spectrometry is used as an analytical technique for metabolite quantification, the marker should be analytically distinguishable from glucose, e.g., in terms of mass-to-charge ratio (*m/z*) or retention time, and also should be distinct from eventually used internal standards.

### Assessment of the source of glucose carryover with lactose as a marker

As we postulated that glucose carryover could possibly originate from the water phase between centrifuged cells or from glucose stuck on the cell surface (Fig. 1), we first investigated the source of glucose carryover using lactose as a carryover marker. To our knowledge, lactose is not produced by budding yeast. Secondly, lactose is a disaccharide, making it distinguishable from d-glucose and the internal standard used for the GC–MS analysis here (i.e., glucoheptose) regarding mass-to-charge ratio and retention time. Finally, *S. cerevisiae* is reported to lack lactose transporters and enzymes for lactose hydrolysis^18^, suggesting that lactose will likely not enter cells nor be assimilated by *S. cerevisiae*.

Indeed, we did not find any increase in cell count after 30 h of culturing *S. cerevisiae* on lactose (Fig. S2), suggesting that this organism indeed cannot grow on lactose as a carbon source. Next, we performed a lactose uptake assay to demonstrate that lactose also cannot be taken up. To this end, yeast cells cultured on glucose were extensively washed with PBS buffer and transferred to a lactose-containing medium. Cells were sampled periodically, thoroughly washed, and quenched. Here, we found that the amount of lactose in the samples was always below the detection limit of GC–MS (Fig. S3), suggesting that – if lactose were transported into the cell – its intracellular concentration would be very low (< 0.14 mM). These two findings together suggest that lactose fulfills criteria (1), (2), and (4) as marker metabolites for carryover. Criterion (3), i.e., whether lactose has the same stickiness as glucose, is unclear. This criterion is vital if the carried-over glucose originates from sticking to the cell wall or membrane (Fig. 1, scenario 2) in contrast to being carried over from only the water phase between cells (Fig. 1, scenario 1).

First, we conjectured that scenario 1 is true, i.e., that carried-over glucose is only from the water phase. In this case, as lactose cannot enter cells, the carried-over glucose amount will be proportional to the lactose amount determined in the cell pellet (*n*_*lac, pellet*_), where the proportionality between the two amounts scales by the ratio between the extracellular glucose concentration (*c*_*glc, ex*_) and the lactose concentrations (*c*_*lac, ex*_) at the time of sampling. We used Eq. 1 to estimate the intracellular glucose concentrations (*c*_*glc, in*_). After subtracting the amount of carried-over glucose from the total glucose determined in the cell pellet (*n*_*glc, pellet*_), the amount of intracellular glucose is then divided by the cell volume (*V*_*tot*_):

1 $${c}_{glc, in}=\frac{1}{{V}_{tot}}\cdot \left({n}_{glc,pellet}-{n}_{lac,pellet}\cdot \frac{{c}_{glc,ex}}{{c}_{lac,ex}}\right)$$

**Figure 3:**
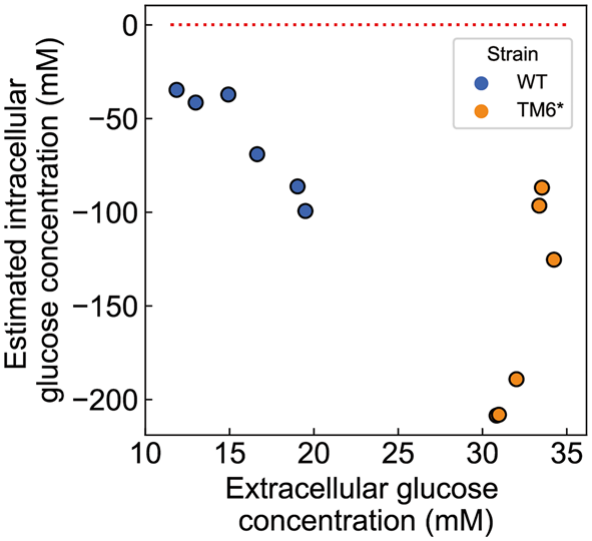
Assessment of the source of glucose carryover using lactose as a marker. Estimated intracellular glucose concentration (mM) vs. measured extracellular glucose concentration (mM). Test of scenario 1, i.e., assuming all glucose carryover comes from the water phase, using lactose as a carryover marker. The red dotted line indicates where intracellular glucose concentration is zero. Six replicates were tested for each strain for the KOY WT strain (blue dots) and KOY TM6* strain (orange dots). Detailed information such as the amount of lactose and glucose determined in the cell pellet and extracellular concentrations of lactose and glucose for each experiment can be found in the supplementary Table S1.

Using this equation together with the respective measurement data from either *S. cerevisiae* KOY wild-type (WT) or the hexose transport-deficient mutant strain TM6*, yet, we obtained negative values for the intracellular glucose concentrations (Fig. 3). This means that the inferred amount of carried-over glucose was larger than the total glucose amount determined in the cell pellet. This suggests that scenario 1 alone, i.e., that the carried-over glucose would just come from the water phase, could not fully explain the carryover effect. Instead, our results suggest that part of glucose carryover also must come from the sticking effect. This implies that a non-glucose molecule is unsuitable as a carryover marker as it likely will have different stickiness compared to glucose.

### Evaluation of the contribution of the sticking effect to the glucose carryover

After finding that glucose carryover not only comes from the water phase (scenario 1) but also must originate from the sticking effect (scenario 2), we then asked how these two scenarios would contribute to the glucose carryover, especially how much glucose carryover originates from the glucose stuck on the cell surface. We reasoned that the amount of glucose carried over could be estimated with a strain that lacks all hexokinase enzymes when cultivated on galactose and glucose. While in fact growing on galactose (due to the lack of all hexokinases), such a strain would still transport glucose into cells via facilitated diffusion. As the imported glucose would not be phosphorylated due to the lack of hexokinases, the intracellular and extracellular glucose concentrations would equilibrate, meaning that the intracellular glucose concentration should be equal to the extracellular one. Assuming equilibrium, the amount of intracellular glucose can then be estimated using the total cell volume (*V*_*tot*_) and the extracellular glucose concentration (*c*_*glc, ex*_). By subtracting the calculated intracellular amount of glucose from the total glucose determined in the pellet (*n*_*glc, pellet*_), one can then estimate the amount of glucose carryover (*n*_*glc, co*_) and normalize it to the total cell surface area (${S}_{tot,hxk-null}$), with the following equation:

2 $${(\frac{{n}_{glc,co}}{S})}_{hxk-null}=\frac{1}{{S}_{tot,hxk-null}}\cdot {n}_{glc, co}=\frac{1}{{S}_{tot, hxk-null}}\cdot \left({n}_{glc,pellet}-{c}_{glc,ex}\cdot {V}_{tot}\right)$$

Following this reasoning, we constructed a *hxk*-null strain, in which the three isozymes of hexokinases (Hxk1, Hxk2, and Glk1) were deleted using CRISPR-Cas9^19,20^. To estimate the glucose carryover per surface area ($\frac{{n}_{glc,co}}{S}$)_*hxk-null*_, we grew the *hxk*-null strain in a minimal medium supplemented with galactose until the mid-exponential phase. We added U-^13^C- d-glucose to a series of cultures to reach different final glucose concentrations. A simulation with a differential equation model, describing the dynamics of glucose important and export, showed that equilibration between the levels of extracellular and intracellular glucose is achieved within around 10 min (calculations in Supplementary Text 1). Based on the simulation results, we chose to take samples three hours after the glucose addition, which we then quenched and processed as described above for lactose assessment.

**Figure 4:**
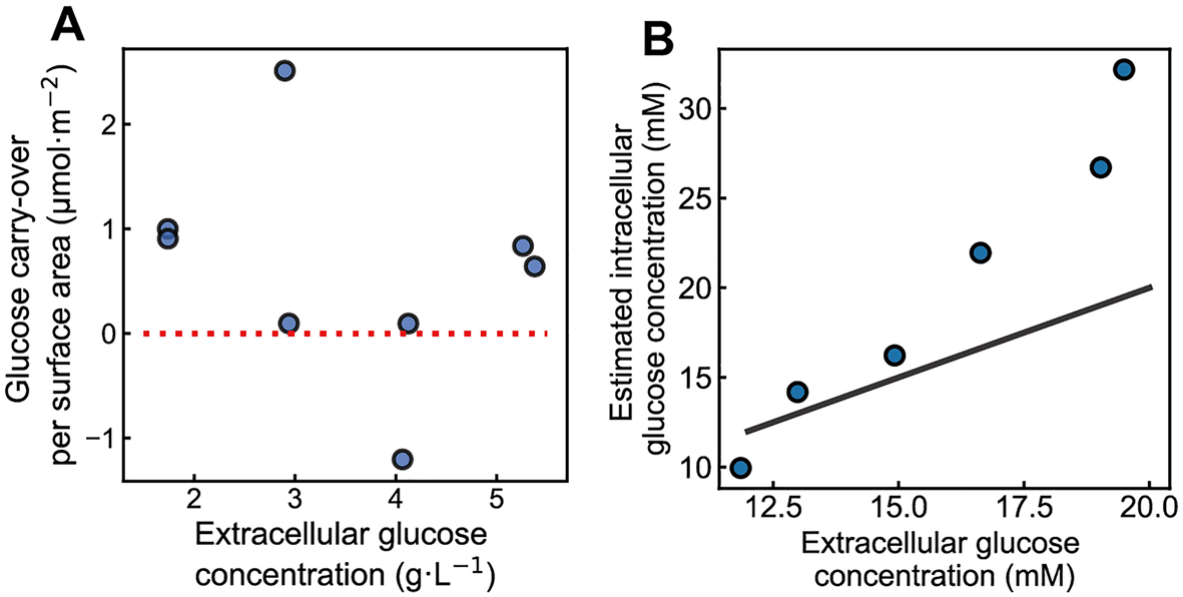
Estimated glucose carryover per cell surface area and intracellular glucose concentrations. (**A**) Estimated glucose carryover per cell surface area at different extracellular glucose concentrations using a hexokinase-deficient strain. The amount of glucose carryover was normalized to the total cell surface area. The red dotted line indicates where the estimated glucose carryover per cell surface area is 0. Two replicates of cultures were taken at each extracellular glucose concentration. (**B**) Estimated intracellular glucose concentration (mM) *vs.* extracellular glucose concentration (mM) using Eq. (3). The extracellular glucose concentrations (from 12 to 20 mM) in Fig. 4B correspond to concentrations of 2.2 g·L^−1^ to 3.6 g·L^−1^ in (**A**). The solid black line indicates where intracellular glucose concentration equals extracellular glucose concentration.

Here, while the data is noisy, we found that the concentration of extracellular glucose seemingly had no impact on the amount of glucose carried over per cell surface area, as there is no clear trend between the extracellular glucose concentration and the amount of glucose per surface (Fig. 4A). This finding suggests that the cell surface must have been saturated already at the lowest extracellular glucose concentration. Averaging all eight independent experiments (except for the negative value), we obtained a glucose carryover per surface area of 0.87 (± 0.81) µmol m^−2^.

It is important to note that the estimated value of glucose carryover per cell surface area could be underestimated. While this value was estimated based on the assumption that intracellular glucose concentration would equilibrate with extracellular glucose concentration, it is also possible that complete equilibration was not achieved in these experiments, for instance, because dilution due to cell growth would occur faster than the glucose transport. Thus, with the assumption of equilibration, we may overestimate the amount of intracellular glucose and thus underestimate the carried-over glucose per cell surface area if ${c}_{glc,ex}>>{c}_{glc,in}$. This means that the estimated value of glucose carryover per cell surface area needs to be seen as a lower limit, and the actual glucose carryover could even be higher than we estimated.

Knowing the glucose carryover per cell surface area, we used the data (i.e., *V*_*tot, WT*_, *n*_*glc, pellet*_) from the lactose addition experiment from above (Fig. 3), determined the total cell surface area of KOY WT (*S*_*tot, WT*_) via microscopy, and used Eq. 3 to estimate intracellular glucose concentrations in the KOY WT strain during the batch culture, with the following equation:

3 $${c}_{glc,in}=\frac{1}{{V}_{tot,WT}}\cdot \left({n}_{glc,pellet}-{S}_{tot,WT}\cdot {\left(\frac{{n}_{glc,co}}{S}\right)}_{hxk-null}\right)$$

Determining the intracellular glucose concentrations in the lactose addition experiment using the above estimated coefficient of glucose carryover per cell surface area, we obtained intracellular glucose concentrations that are higher than the extracellular ones (Fig. 4B), suggesting that glucose carryover per surface area we estimated here is an underestimation.

Still, with the above estimated value, assuming glucose molecules would uniformly spread on the cell surface, we calculated the number of layers of glucose molecules on the surface of a single cell based on the theoretical size of a glucose molecule, the surface area of a cell, and the estimated glucose carryover per surface area (cf. calculations in the Supplementary Text 2). Here, we found that carried-over glucose molecules would correspond to approximately 30 layers on the surface of a cell. This calculation suggests that most glucose carryovers must primarily come from the water phase (scenario 1), as glucose molecules may not stack so many layers on the surface of a cell. Given the fact that we likely underestimated the glucose carryover per cell surface area and the actual number of ‘stacking layers’ of glucose carryover would be greater than 30, we believe that the majority of glucose carryover must originate from the water phase, rather than from the sticking effects.

To conclude, using a hexokinase-deficient strain, we estimated glucose carryover per cell surface area based on the assumption that intracellular glucose could equilibrate with extracellular glucose in this strain. With the calculated number of layers of glucose molecules on the cell surface, we found that glucose stuck on the cell surface only contributes a minor fraction to the glucose carryover. At the same time, the majority still originates from the water phase sampled with the cell pellet.

### Dynamics of l-glucose uptake in budding yeast

Previously, l-glucose was used as a carryover marker when determining intracellular glucose concentrations^1^. As the enantiomer of d-glucose, l-glucose likely has the same stickiness to the cell surface. This is important since, as we have shown above, the carryover originates not only from the water phase but also from glucose molecules sticking to the cell surface. In the previous study where l-glucose was used as a carryover marker, it was assumed that this metabolite was not taken up^1^. However, there have also been reports showing that l-glucose could be transported into yeast cells^13,14^, making l-glucose thus a questionable carryover marker.

Even if l-glucose is taken up, it could still be used as a marker if the uptake dynamics are very slow such that in the time between adding it to the medium and taking the sample, no significant amount of l-glucose would have ended up inside cells. To this end, we next determined the rate of l-glucose uptake, for which we first had to develop a method to distinguish l- and d-glucose analytically. Here, instead of using the radioactive labeled d-[^14^C]-glucose and l-[^3^H]-glucose, as has been done in the past^1^, we used mass spectrometry and the uniformly ^13^C-labeled version of d-glucose -^13^C_6_ and non-labeled l-glucose. While these two glucose molecules after the derivatization have the same retention time in the used gas chromatography column, their different mass-to-charge ratios after fragmentation in the GC–MS (labeled d-glucose: 323, l-glucose: 319) allowed us to separate them in the mass spectrometer.

To determine the l-glucose uptake dynamics, we cultivated a wild-type yeast strain (*S. cerevisiae* KOY WT) on a minimal medium with ^13^C-labeled d-glucose in three consecutive overnight pre-cultures to ensure cells were fully ^13^C-labeled. After washing the cell pellets twice thoroughly with the PBS buffer to remove any glucose as much as possible, we added the cell suspension to aliquots of media supplemented with unlabeled l-glucose and incubated the cells for different periods of time. At different time points, we quenched the cells of an aliquot and extracted intracellular metabolites.

**Figure 5:**
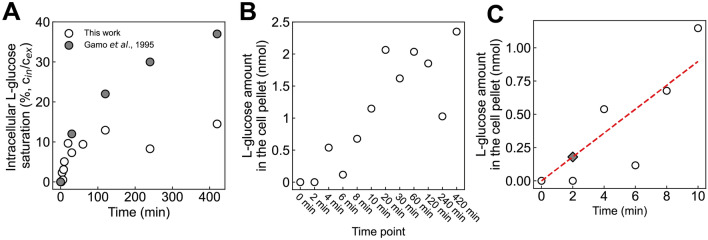
l-glucose could slowly enter cells. (**A**) Percentage of saturation of intracellular glucose concentration (% of extracellular l-glucose concentration). open circles are data points from this work, while closed grey circles are from a previous report^14^. (**B**) The amount of l-glucose (in nmol) determined in the cell pellet of each sampling time point. Open circles represent amounts of l-glucose measured by the GC–MS from 0 to 420 min. (**C**) The amount of l-glucose determined in the cell pellet within the first ten minutes of the l-glucose uptake assay. Open circles represent amounts of l-glucose measured by the GC–MS from 0 to 10 min. A linear regression line (red dashed line) was determined to estimate the dynamics of intracellular l-glucose accumulation within 10 min (*y* = 0.0897*x*, *R*^2^ = 0.8524). The closed diamond represents the estimated amount of intracellular l-glucose after two minutes of adding l-glucose to the cell culture, following the estimated linear accumulation.

Consistent with previous reports^13,14^, we found that l-glucose could enter cells (Fig. 5A). After seven hours, the intracellular l-glucose concentration reached a steady level of around 12% of the extracellular l-glucose concentration (Fig. 5A). In a previous study, a 40% saturation was achieved^14^, possibly due to the 10 times higher l-glucose concentration used in this study (100 mM). Notably, at the two-minute timepoint, we could not yet detect any l-glucose (Fig. 5B). We considered this to be due to the detection limit (< 0.1 nmol). Assuming a linear buildup of l-glucose over the first 10 min, we estimated the actual amount of l-glucose at this time point (Fig. 5C).

These experiments demonstrated that l-glucose can enter cells. Thus, if l-glucose is to be used as a marker for the carryover, it should be added only shortly before the actual sampling, and the amount of l-glucose already entered during even a short period of time, has likely to be considered as well.

### Determining intracellular glucose concentration with dynamic addition of l-glucose as the marker

Using l-glucose as a carryover marker, we then aimed to determine the intracellular glucose concentrations in two yeast strains: KOY WT and TM6*. Specifically, we spiked l-glucose into cultures exponentially growing on U-^13^C- d-glucose two minutes before we quenched the samples to determine the intracellular glucose concentration.

To calculate the amount of intracellular l-glucose, we multiplied the total cell volume (*V*_*tot*_) with the estimated intracellular l-glucose concentration at 2 min (*c*_*L-glc,in*_). Then we subtracted the amount of intracellular l-glucose from the total l-glucose determined in the cell pellet (*n*_*L-glc,pellet*_) to calculate the amount of l-glucose carryover. Since both parts of carried-over glucose from the two scenarios (i.e., from the water phase or sticking) scale to their extracellular concentrations, we can then infer the amount of carried-over d-glucose from the amount of l-glucose carryover and the extracellular concentration ratio between d-glucose (*c*_*D-glc, ex*_) and l-glucose (*c*_*L-glc, ex*_). By subtracting the carried-over d-glucose from the total d-glucose amount that we determined in the cell pellet (*n*_*D-glc, pellet*_), we then can calculate the intracellular d-glucose concentration (Eq. 4) by dividing the amount of inferred intracellular d-glucose with the total cell volume (*V*_*tot*_):

4 $${c}_{D-glc, in}=\frac{1}{{V}_{tot}}\left[{n}_{D-glc, pellet}-\frac{{c}_{D-glc,ex}}{{c}_{L-glc,ex}}\cdot \left({n}_{L-glc,pellet}-{c}_{L-glc,in}\cdot {V}_{tot}\right)\right]$$

**Figure 6:**
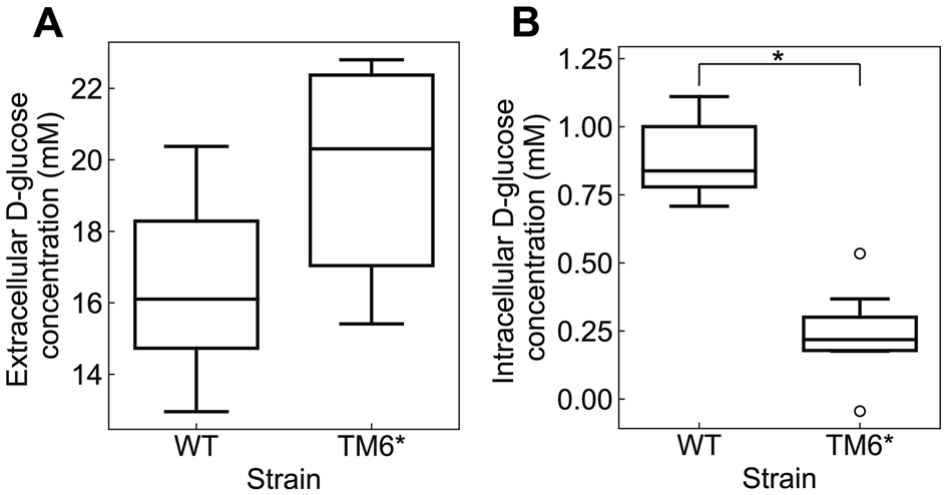
Extracellular glucose concentrations and estimated intracellular glucose concentrations in KOY wild-type and TM6* strain. (**A**) Distributions of extracellular d-glucose concentrations in KOY wild-type and TM6* strain at the moment of sampling for the intracellular glucose concentrations. (**B**) Distributions of estimated intracellular d-glucose concentrations in KOY wild-type and TM6* strain after subtracting intracellular l-glucose from the total amount of l-glucose in the cell pellet. Seven replicates were taken for each strain. Open circles represent the outliers of the box plot. Student’s t-test was performed for intracellular glucose concentrations between the wild-type strain and mutant strains, and the asterisk indicates the statical significance *p*-value was lower than 0.001.

With this equation and the respective measurement data, we then estimated the intracellular d-glucose concentrations in both KOY wild-type and KOY TM6* when cells were grown on U-^13^C- d-glucose and adding l-glucose to a final concentration of 1 g·L^−1^ two minutes before quenching. All samples were collected during the exponential growth phase when the extracellular glucose concentration had values between 12.95 to 22.80 mM (2.33 to 4.11 g·L^−1^, Fig. 6A). Here, we found a significant difference (*p*-value < 0.001) in intracellular glucose concentrations between the wild-type and TM6*, with estimated mean values of 0.89 mM for the wild-type strain and 0.24 mM for the TM6* strain respectively (Fig. 6B). Intracellular glucose concentrations estimated for the wild-type strain (0.89 mM) are comparable to the measurements for the wild-type (1.5 mM) as previously reported^1^.

## Discussion

What are intracellular glucose concentrations in yeasts when yeast is grown on glucose? Despite the importance of intracellular glucose levels for glucose sensing and transport, we know little about their levels due to the analytical challenge caused by the carryover of glucose from the medium. In this study, we found that carried-over glucose originates from both the water phase and the glucose sticking to the cell surface. However, the sticking part only contributes to a minor fraction of the glucose carryover. Based on the fact that l-glucose is structurally close to d-glucose, and is transported into cells while not being metabolized, we eventually used it as a carryover marker to correct for the carryover effect and estimate intracellular glucose concentrations in two yeast strains with distinct kinetics of glucose transport.

In this study, we evaluated two molecular markers for the glucose carryover effect. Although lactose fulfills almost all requirements as a carryover marker such as being distinguishable from d-glucose with mass-spectrometry, non-assimilative by the baker’s yeast, etc., we still cannot use it as a carryover marker due to its unknown stickiness to the cell wall (or cell membrane) compared to d-glucose. It should be noticed that even though we found that glucose molecules stuck on the cell surface only contribute to a minor extent to the carryover effect, the disaccharide lactose may behave differently from monosaccharide D- or l-glucose on the cell surface due to its larger molecular size, making it impossible to use it to correct for the carryover effect. Eventually, we revisited l-glucose as the carryover marker. Although this molecule can be slowly taken up by yeasts, l-glucose is still the most suitable marker to correct for the glucose carryover effect that we found so far.

As l-glucose slowly enters cells, here, we corrected for the small amount of l-glucose that had entered during the two minutes after its addition. But was it necessary to do so? Assuming that after two minutes no l-glucose had entered, we would have obtained negative values of intracellular glucose concentration of -0.09 mM for the TM6* strain (Fig. S4). In contrast, when estimating the intracellular l-glucose concentration at 2 min with a linear regression of accumulated l-glucose across the first ten minutes (Fig. 5C), we obtained positive values of intracellular glucose concentrations for both KOY WT and TM6* (Fig. 6B). This suggests that when using l-glucose as a carryover marker and even when added shortly before the sampling, it is important to determine its uptake dynamically and to correct for the even little amount that has been taken up.Ideally, the extracellular glucose in the water phase and the one sticking to cells would be washed away during the processing of the cell samples. However, such processing inherently requires quenching of any cellular enzymatic activity to preserve the endogenous intracellular metabolite levels, including the one of intracellular glucose. As turnover rates in the primary metabolism of microbes are typically in the sub-second to second range, quenching has to be extremely fast. To this end, -40 °C cold methanol is typically used as a quenching agent. However, the solubility of glucose in the cold methanol is poor^21^, and therefore extracellular glucose might only insufficiently be removed. If one opts to apply several consecutive washing steps with -40 °C cold methanol, the more extensive exposure to the organic solvent could lead to leakage of metabolites from cells^9,22^, which again would impact the intracellular metabolite levels. While other quenching agents with improved glucose solubility could potentially reduce the glucose carryover effect, we are doubtful that a compound could be found that fulfills this and all other requirements for an effective quenching agent. Thus, we feel that if intracellular glucose levels need to be accurately measured, when cells are grown on glucose, one likely needs to apply methods as we propose here.

In summary, our study demonstrates the persistent difficulty in accurately measuring intracellular glucose levels when glucose is used as a substrate in the medium. Beyond previous works, we provide insights into the origin of glucose carryover and the distribution of glucose carryover from different parts: the contribution of the sticking effect of the overall carryover effect is limited while a significant source of the glucose carryover should originate from the water phase. Then, we chose l-glucose as a marker to correct the carryover effect, which requires a careful assessment of its dynamic uptake via experiments before applying it as a carryover marker. Nevertheless, since we used a pair of non-radioactive glucose (both configurations) compared to the radioactive pair used in the previous study^1^, we could see a broader application of our method practiced in labs with stricter environmental restrictions. We eventually estimated intracellular glucose concentrations for two yeast strains with markedly distinct kinetics of glucose uptake. Considering the role of intracellular glucose as a critical signaling molecule, these differences emphasize the importance of accurate measurements.

## Material and methods

### Construction of the hexokinase-null strain

The hexokinase-null (*hxk*-null) mutant was constructed on the S288C-derived prototrophic YSBN6 strain (*MATa* *ho::HphMX4*)^23^ via CRISPR-Cas9 using a MoClo Yeast Toolkit^19,20^. Target sequences and repair fragments used for CRISPR-Cas9 deletions can be found in Table S2. The final strain (YSBN6 ∆*hxk1*∆*hxk2*∆*glk1*) was verified by PCR and Sanger sequencing.

### Yeast strains and maintenance

Unless specified, all chemicals were purchased from Sigma-Aldrich. Before the main experiment, glycerol stocks of the prototrophic *S. cerevisiae* KOY PK2-1C83 (relevant genotype: wild-type, *MATa* *MAL2-8*^*c*^* SUC2*) strain and its hexose transport-deficient mutant TM6* (relevant genotype: chimeric *HXT1*-*HXT7* glucose transporter as sole hexose transporter)^17^ were restreaked on the YPD-agar plates (1% [w/v] yeast extract, 2% [w/v] peptone, 2% [w/v] glucose, 1.5% [w/v] agar) and cultivated at 30 °C to obtain single colonies. The *S. cerevisiae* YSBN6 hxk-null strain was restreaked on the YPG-agar plates (1% [w/v] yeast extract, 2% [w/v] peptone, 2% [w/v] galactose, 1.5% [w/v] agar) and cultivated at 30 °C till the single colonies appear. All plates were freshly prepared and restreaked less than one week before each experiment.

### Shake flask cultivation

The minimal medium^24^ was used in all batch cultures in this work, supplemented with different carbon sources at varied concentrations. The minimal medium (pH 5.0) was always prepared freshly (< 24 h) before the experiment to avoid any possible salt precipitation. All batch cultivations were performed in 100 mL-shaking flasks (filled with 10 mL of the medium) at 30 °C with a shaking speed of 300 rpm in a shaking incubator (Kuhner AG).

To investigate the source of glucose carryover using lactose as a marker, single colonies of KOY WT and TM6* from the YPD agar plates were inoculated individually in 10 mL of minimal medium (10 g·L^−1^ glucose) and cultivated for eight hours. This first batch of pre-culture was then diluted into another 10 mL of minimal fresh medium (10 g·L^−1^ glucose) as the second batch of pre-culture to reach a cell density OD_600_ of 1 to 2 (equivalent to the cell density during the mid-exponential phase) after the overnight cultivation. The cell density OD_600_ was measured by a spectrophotometer (Novaspec Plus). The overnight pre-culture was then diluted to preheated 10 mL of minimal medium (6 g·L^−1^ glucose and 1 g·L^−1^ lactose) with a starting cell density OD_600_ of 0.15 (WT) and 0.2 (TM6*) separately. For each strain, three replicates were inoculated and sampled during the steady state, and another three replicates were repeated on another day.

To evaluate whether l-glucose can be used as a glucose carryover marker, single colonies of KOY WT and TM6* from the plates were inoculated individually in 10 mL of minimal medium supplemented with 10 g·L^−1^ uniformly ^13^C-labeled d-glucose (CAS: 110187–42-3, Sigma-Aldrich). The first overnight culture was diluted into two consecutive batches of overnight cultures to ensure cells were already fully ^13^C-labeled before the main cultures. After the third pre-culture, labeled cells were diluted into preheated 10 mL minimal medium (6 g·L^−1^ U-^13^C- d-glucose) with a starting cell density OD_600_ of 0.15 (WT) and 0.2 (TM6*) separately in order to collect cells at comparable cell densities for two strains (as TM6* grows slower than the WT strain). l-glucose (CAS: 921–60-8, Sigma-Aldrich) was added into the culture as a glucose carryover marker two minutes before sampling with a final concentration of 1 g·L^−1^ in the culture. For each strain, six replicates were performed and sampled during the steady state.

To estimate glucose carryover per cell surface area using the *hxk*-null strain, single colonies of YSBN6 *hxk*-null from the YPG agar plates were inoculated in 10 mL of minimal medium (10 g·L^−1^ galactose). Following the pre-culture protocol for assessing lactose as a glucose carryover marker while using galactose as the carbon source, the overnight pre-culture was diluted into preheated 10 mL of minimal medium (10 g·L^−1^ galactose) with a starting OD_600_ of 0.15. To allow intracellular glucose to equilibrate with glucose in the medium, glucose was added to the exponentially growing culture three hours before sampling to a final concentration of 2 to 5 g·L^−1^, respectively. Two parallel cultural replicates were sampled for each extracellular glucose concentration.

### Rapid sampling with cold methanol quenching and boiling ethanol extraction

To determine intracellular metabolite levels, cells were quenched, and intracellular metabolites were extracted as previously described^15,25^. Specifically, an aliquot of 7 mL of mid-exponential phase culture (approximately 10 OD_600_) was quenched by spiking the culture into a 50-mL prechilled Falcon tube filled with 35 mL of cold methanol (-40 °C). The quenched cell pellet was then filtrated on a biomass filter (Supor-200, 0.2 µm, 47 mm, Pall Corporation), which was pre-washed with 15 mL of cold pure methanol before collecting quenched cell pellets. The filter containing the quenched cell pelleted was washed with 15 mL of cold methanol and transferred into 30 mL of preheated 75% (v/v) ethanol solution. An aliquot of 500 µL of 500 µM glucoheptose (CAS: 62475–58-5, Sigma-Aldrich) solution was added into the extraction solution as an internal standard (ISTD), and the cell pellet was extracted at 95 °C for 3 min. To further concentrate extracted metabolites, the extraction solution was dehydrated with a centrifuge concentrator (Concentrator plus, Eppendorf) at 30 °C overnight. The dried sediment in the dehydrated samples was re-constituted in 500 µL of Milli-Q water. After centrifuging at 17,949 × *g* for 5 min at 1 °C, the supernatant was transferred to a new 1.5-mL Eppendorf tube and repeated the centrifuging step to remove the cell debris or protein precipitate as much as possible. Following the step centrifuging step, the supernatant was collected and stored at −70 °C until further analysis.

To determine the concentrations of extracellular metabolites, an aliquot of 500 µL of culture was collected and filtrated with Spin-X column filters (0.22 µm pore size, Corning). The culture supernatant was diluted 50 times with Milli-Q water (as concentrations of metabolites in the culture medium were too high for the GC–MS analysis) and processed as same as intracellular samples from the point of adding the internal standard and extraction at 95 °C for 3 min.

### Analysis of metabolites using the gas chromatography-mass spectrum (GC–MS)

To quantify the amounts of metabolites in either the cell pellet or the medium, an aliquot of 50 µL of concentrated samples was transferred to a 1.5-mL Eppendorf tube and dehydrated at 30 °C for the derivatization reactions in the organic phase. Subsequently, the dried sample was resuspended with 50 µL of 20 mg·mL^−1^ methoxylamine hydrochloride (CAS: 593–56-6, Supelco) solution (dissolved in pyridine freshly before derivatization). After the incubation at 70 °C for 50 min, samples were further derivatized via reacting with 80 µL of MSTFA (standard concentration: 98–100%) plus 1% TMCS (TS-48915, Thermo Fisher Scientific) at 70 °C for another 50 min. An aliquot of 90 µL of supernatant of derivatized samples was collected after centrifuging at 10,621 × *g* for 2 min.

Identification and quantification of sugars were implemented using a gas chromatography system coupled to a single quadrupole mass spectrometer (GCMS-QP2010 Ultra, Shimadzu). An aliquot of 1 µL of the derivatized sample was injected into a GC column (VF-17 ms GC column, 30 m, 0.25 mm, 0.25 µm, 7-inch cage, Agilent) at 300 °C at a split ratio of 1:20 using helium as the carrier gas with a flow rate of 1 mL·min^−1^. The GC oven temperature was kept at 70 °C for 5 min and gradually increased to 260 °C with a ramp of 6 °C·min^−1^, held for 10 min. The ion source temperature of the mass spectrometer was set to 200 °C, with an interface temperature of 300 °C. The mass spectrum was acquired using the selected ion monitoring (SIM) mode with a detector voltage of 0.9 kV. Target compounds and their mass-to-charge ratios (m/z) are listed in Table S3.

Calibration curves were established for all target metabolites using standard samples with known concentrations. A series of standard samples were prepared by dissolving the compound in water and serially diluted into standard samples with lower concentrations. The concentration range covered by the standard samples is listed in Table S3 for each compound. To prepare the calibration curve for each compound, an aliquot of 500 µL of each standard sample was mixed with 500 µL of 500 µM glucoheptose solution, derivatized, and injected into the GC–MS as described above. The ratio of the integrated peak area between the target compound (e.g., glucose, lactose) and the internal standard (glucoheptose) at each concentration was calculated. Then the ratios of the area were plotted against the already known concentrations, and the calibration curves were fitted using quadratic functions. With the calibration curves of each compound, the concentrations of the target compounds in the cell extract and the supernatant of the culture were analyzed with the software of GCMSsolution (version 4.11) from Shimadzu.

### Lactose growth test

To test whether the prototrophic *S. cerevisiae* strain can grow on lactose, single colonies of the KOY WT strain were first cultivated overnight in 10 mL of minimal medium with 10 g·L^−1^ galactose till the mid-exponential phase. The overnight culture was centrifuged at 3,214 × *g* at 20 °C for 5 min to remove residual galactose in the supernatant, and the cell pellet was washed twice with 1 mL of PBS buffer (pH = 7.4) in a sterile Eppendorf tube. The washed cell pellet was resuspended in 50 mL minimal medium supplemented with 5 g·L^−1^ lactose. Growth of the KOY wild-type strain in the lactose culture was followed by measuring cell counts periodically using a flow cytometer (Accuri C6, BD Biosciences).

### Lactose uptake assay

To assess whether lactose could be transported into cells, single colonies of KOY WT were inoculated into 10 mL of minimal medium containing 10 g·L^−1^ glucose. After eight hours of cultivation, the culture was diluted in a fresh 10 mL minimal medium (10 g·L^−1^ glucose) and propagated overnight to reach a cell density OD_600_ of 2 the following day. To remove glucose in the culture, the overnight pre-culture was centrifuged at 3,214 × *g* for three minutes to discard the supernatant. The cell pellet was washed twice with 10 mL of PBS buffer (pH = 7.4) and resuspended in 50 mL preheated minimal medium (5 g·L^−1^ lactose). At each sampling time, an aliquot of 4 mL culture was sampled from the culture and centrifuged at 3,214 × *g* for three minutes to remove the supernatant. After washing the cell pellet with 1 mL of PBS buffer, samples were quenched with 5 mL of cold methanol (-40 °C) and extracted following the protocol above to extract intracellular metabolites. Samples were taken from two independent cultures every two hours at each time point.

### l-glucose uptake assay

To test whether l-glucose could enter cells and, if yes, how fast it would happen, single colonies of KOY WT were inoculated in 10 mL minimal medium (10 g·L^−1^ U-^13^C- d-glucose) and propagated via three consecutive batches of overnight pre-cultures to ensure cells were fully ^13^C-labeled. The third batch of pre-culture was centrifuged at 3,214 × *g* for three minutes to remove the supernatant, and the remaining cell pellet was washed twice with 10 mL of the PBS buffer. The washed cell pellet was resuspended in 3 mL of preheated minimal medium (without any carbon source) and incubated at 30 °C. An aliquot of 200 µL of concentrated cell suspension was added to 4 mL of minimal medium with 2 g·L^−1^ l-glucose and cultivated at 30 °C for different periods (0 min, 2 min, 4 min, 6 min, 8 min, 10 min, 20 min, 30 min, 1 h, 2 h, 4, h, and 7 h). At each time point, an aliquot of 4 mL culture was taken and centrifuged at 3,214 × *g* for three minutes to remove the supernatant. The cell pellet was washed with 1 mL of the PBS buffer, then quenched with 5 mL of cold methanol (-40 °C), and metabolites were extracted.

### Quantification of the total cell volume and total cell surface area

The total cell volume of each sample was determined by a cell counter and analyzer CASY (OLS OMNI Life Science) using both the cell count and cell size distributions. Shortly before sampling for intracellular metabolites, an aliquot of 20 µL of mid-exponential growing culture was thoroughly mixed with 10 mL of CASY TON solution and subsequently analyzed by the CASY device. The CASY device returns cell numbers (*n*_*d*_) measurements in each size channel with different diameters (*d*). The total cell volume (*V*_*tot*_) was calculated by integrating cell volumes in each diameter channel from 0 to 20 µm and amplified by multiplying a dilution constant *D* (20 µL in 10 mL) and the volume of each sample *V*_*Sample*_ (Eq. 5).

5 $${V}_{tot}={V}_{Sample}\cdot D\cdot {\sum }_{0}^{20}{n}_{d}\cdot {\frac{4}{3}\cdot \pi \cdot \left(\frac{d}{2}\right)}^{3}$$

To quantify each sample's total cell surface area, an aliquot of 2 µL of steady-state culture was collected. Images of cells were captured by an inverted fluorescence microscope (Eclipse Ti-E; Nikon Instrument) with a 100 × oil immersion objective and analyzed using an image processing program ImageJ (1.48v) and the plugin BudJ (v4.3) as described previously^26^. For each strain, samples from three flasks were collected, and around 100 cells in each replicate were analyzed in terms of surface area, cell volume, and surface-to-volume ratio. Each strain's surface-to-volume ratio [${(\frac{S}{V})}_{Mean}$] was determined by averaging the mean surface-to-volume ratio from triplicates. The total cell surface area was calculated by multiplying the average surface-to-volume determined via microscopy with the total cell volume determined via the CASY device (Eq. 6).

6 $${S}_{tot}={V}_{tot}\cdot {\left(\frac{S}{V}\right)}_{Mean}$$

### Supplementary Information

Supplementary Information.

## Data Availability

All data generated or analyzed during this study are included in this published article (and its Supplementary Information files).
