# Supplementary material for: Quantifying intracellular glucose levels when yeast is grown in glucose media
Source: Sci Rep. 2023 Oct 10;13:17066. doi: 10.1038/s41598-023-43602-z (PMC10564791; doi:10.1038/s41598-023-43602-z)
Supplement: Supplementary file 1 — Supplementary Information. [file 41598_2023_43602_MOESM1_ESM.docx]

**Supplementary Information**

**
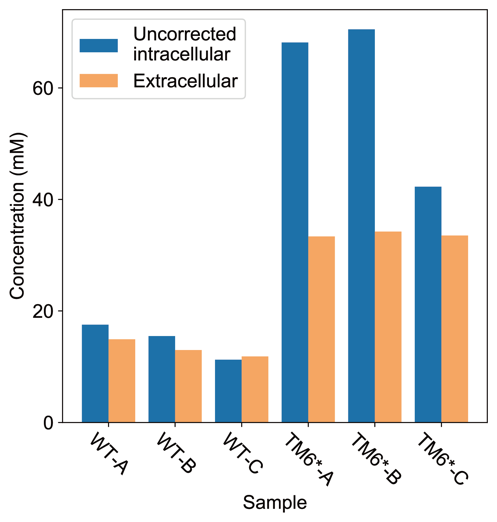
**

**Fig. S1**: Comparison between the extracellular glucose concentration in the medium and ‘intracellular’ glucose concentrations if no correction for carryover is applied. Uncorrected intracellular glucose concentrations were calculated by dividing all glucose amounts determined in the cell pellet (including both intracellular glucose and carried-over glucose) by the total cell volume sampled in each sample. The blue bars indicate the uncorrected ‘intracellular glucose concentrations, while the orange bars indicate the extracellular glucose concentrations. A, B, and C indicate three independent cultures for each strain.

**
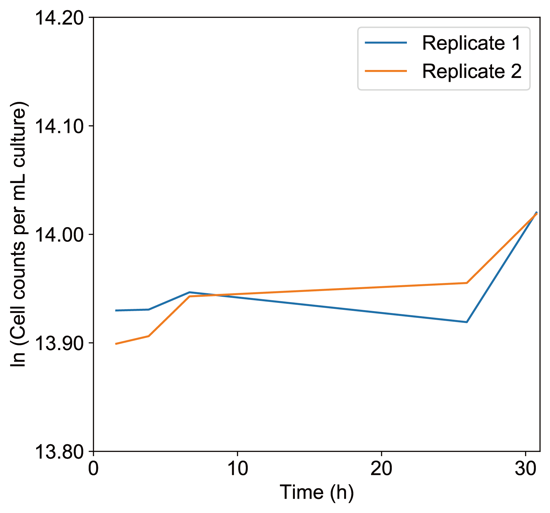
**

**Fig. S2:** Growth curve of *S. cerevisiae* KOY WT in the minimal medium containing 10 g·L^-1^ lactose at 30°C. Two single colonies were pre-cultured in the minimal medium supplemented with 10 g·L^-1^ galactose. The cell pellet from the two cultures was thoroughly washed and diluted in the lactose medium. The evaporation of the medium probably introduced a slight increase at the end of the culture during the culture.


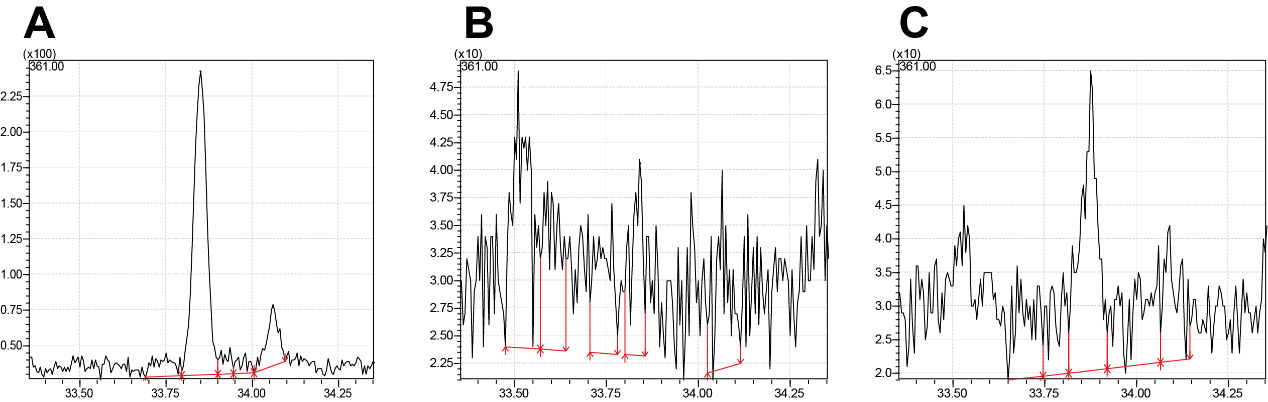


**Fig. S3: Chromatogram of lactose at a mass-to-charge ratio of 361 during the lactose uptake assay**. (A) Peaks of lactose at *m*/*z* =361 of the standard sample with the lowest concentration. A clear and sharp peak of lactose could be found in this standard sample at the retention time of 33.85 min with a concentration of 2.42 nM (corresponding to 0.14 mM intracellular concentration given the total cell volume of 8.8 µL at the first time point of sampling). (B) Peaks of lactose at *m*/*z* = 361 of the first time point during the lactose uptake assay (0 min). The sample was collected immediately after adding lactose to the culture medium. (C) Peaks of lactose at *m*/*z* = 361 of the last time point (8 hours). Compared to the standard sample with the lowest concentration (A), signals of lactose in both (B) and (C) were lower and close to the level of background noise, suggesting that even if lactose could be transferred into cells, levels of intracellular lactose would be extremely low and ultimately close to zero.


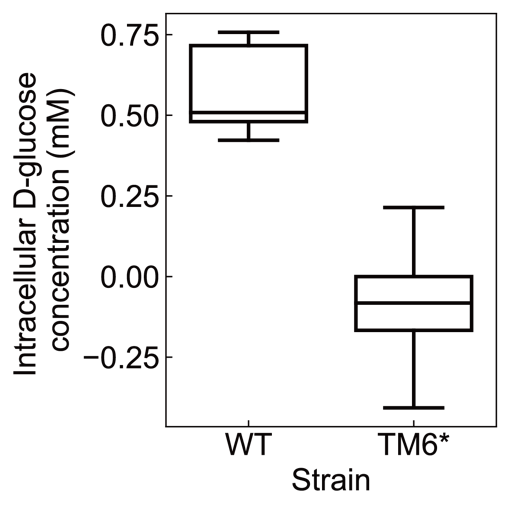


**Fig. S4: Distributions of estimated intracellular D-glucose concentrations in KOY wild-type and TM6* strain assuming no L-glucose has entered cells within the two minutes**. Cells were grown to the mid-exponential phase for each strain in uniformly ^13^C-labeled D-glucose, and non-labeled L-glucose was added to the culture two minutes before quenching. Intracellular D-glucose concentrations of two strains were inferred based on two assumptions: (1) no L-glucose presents intracellularly within the two minutes; (2) Within these two minutes, L-glucose fully equilibrates with the cell wall/membrane. Seven replicates were sampled for each strain.

**Table S1. The amount of lactose and glucose determined in the cell pellet when using lactose as a glucose carryover marker**

| **Sample** | **Lactose in the cell pellet (µmol)** | **Glucose in the cell pellet (µmol)** | **Extracellular glucose concentration (mM)** | **Extracellular lactose concentration (mM)** |
| --- | --- | --- | --- | --- |
| WT-Rep-1 | 0.12 | 0.18 | 14.92 | 3.19 |
| WT-Rep-2 | 0.13 | 0.15 | 12.99 | 3.16 |
| WT-Rep-3 | 0.12 | 0.11 | 11.85 | 3.13 |
| WT-Rep-4 | 0.14 | 0.27 | 19.50 | 2.63 |
| WT-Rep-5 | 0.13 | 0.23 | 19.03 | 2.68 |
| WT-Rep-6 | 0.14 | 0.22 | 16.63 | 2.67 |
| TM6*-Rep-1 | 0.11 | 0.57 | 33.37 | 2.59 |
| TM6*-Rep-2 | 0.13 | 0.58 | 34.24 | 2.66 |
| TM6*-Rep-3 | 0.10 | 0.42 | 33.53 | 2.66 |
| TM6*-Rep-4 | 0.15 | 0.40 | 30.84 | 2.78 |
| TM6*-Rep-5 | 0.14 | 0.39 | 30.97 | 2.79 |
| TM6*-Rep-6 | 0.13 | 0.35 | 32.02 | 2.94 |

**Table S2. List of target sequences and repairing fragments used in this study**

| **Name** | **Sequence (5’ to 3’)** |
| --- | --- |
| The target sequence of Hxk1 | GTTATAGACAGAACCGTCAG |
| The target sequence of Hxk2 | CTTAGATTGAGTGGTGTCAA |
| The target sequence of Glk1 | GAGAGCGGTCATCCAGGCCG |
| Repair fragment for ∆*hxk1* | AATTCTTTTCTTTTAATCAAACTCACCCAAACAACTCAATTAGAATACTGAAAAAATAAGTGAAAAAAATGTAATGAAATATAAATGTGTTTTTCCCTCCCTTAATATTATTATTCTTAT |
| Repair fragment for ∆*hxk2* | CTTTGAAAAGGTTGTAGGAATATAATTCTCCACACATAATAAGTACGCTAATTAAATAAAACTTAATTTGTAAATTAAGTTTGAACAACAAGAAGGTGCCCTTTTTCTACTTATGTGAAC |
| Repair fragment for ∆*glk1* | GCCCAACTCAGCTTCCGTAAACCACAACACCACCACTAATACAACTCTATCATACACAAGTCTTTTTACATTTTTTTGGTTTGTGTACGTATCCCACCGTACTTACCATCTTCTCTCCTT |

**Table S3. List of target compounds: mass-to-charge ratios after the derivatization and the range of concentrations in the derivatized samples covered by the standard samples.**

| Compound | Mass-to-charge ratio after fragmentation in GC-MS (*m*/*z*) | Concentrations of standard samples (µM) |
| --- | --- | --- |
| Non-labeled D-glucose | 319 | 10 to 2000 |
| Non-labeled L-glucose | 319 | 10 to 2000 |
| U-^13^C_6_-D-glucose | 323 | 10 to 2000 |
| Lactose | 361 | 2 to 200 |
| Glucoheptose | 331 | * |

* Glucoheptose was used as the internal standard, so its calibration curve was unnecessary.

**Supplementary Text 1: Simulation of equilibration dynamics of the intracellular glucose concentrations in the *hxk*-null strain**

Since glucose transport is a symmetric process using the same set of hexose transporters, to estimate the equilibration dynamics of the intracellular glucose concentrations, we used the kinetic parameters of glucose transport for the parental strain of the *hxk*-null strain YSBN6. The *V_max_* for glucose transport of YSBN6 is 15.02 mmol·gDW^-1^·h^-1^, and the *K_m_* is 22.96 mM (unpublished data). Also, we determined its biomass concentration (*c_biomass_*) to be 450 gDW·L^-1^ (cell volume). Then, we set up the following ordinary differential equation (ODE) model based on Michaelis-Menten kinetics, assuming a constant extracellular glucose concentration:

The rate of glucose influx (from the extracellular environment to the cytosol):

$$\begin{aligned} r_{influx}=\frac{V_{max}\cdot c_{glc,ex}}{K_{m}+c_{glc,ex}}\#\left( \mathrm{SI}.1 \right) \end{aligned}$$

The rate of glucose efflux (from the cytosol to the extracellular environment):

$$\begin{aligned} r_{efflux}=\frac{V_{max}\cdot c_{glc,in}}{K_{m}+c_{glc,in}}\#\left( SI.2 \right) \end{aligned}$$

Since *V_max_* is normalized to the cell dry weight, we need to convert it to the cell volume using the *c_biomass_*. The change of intracellular glucose concentration with time is

$$\begin{aligned} \frac{{dc}_{glc,in}}{dt}={(r}_{influx}-r_{efflux})\cdot c_{biomass}\#\left( SI.3 \right) \end{aligned}$$

We then integrated the ODE starting from the initial conditions that the initial intracellular glucose concentration is zero. This this end, we used the ODE integration function (odeint) from a Python package Scipy. Since we measured the glucose carryover using the *hxk*-null strain under the extracellular glucose concentrations from 12.95 mM to 22.80 mM, we simulated two extreme conditions for extracellular glucose concentrations 10 mM and 25 mM. Here, we found that intracellular glucose concentrations equilibrates within roughly 10 min (see Figures).


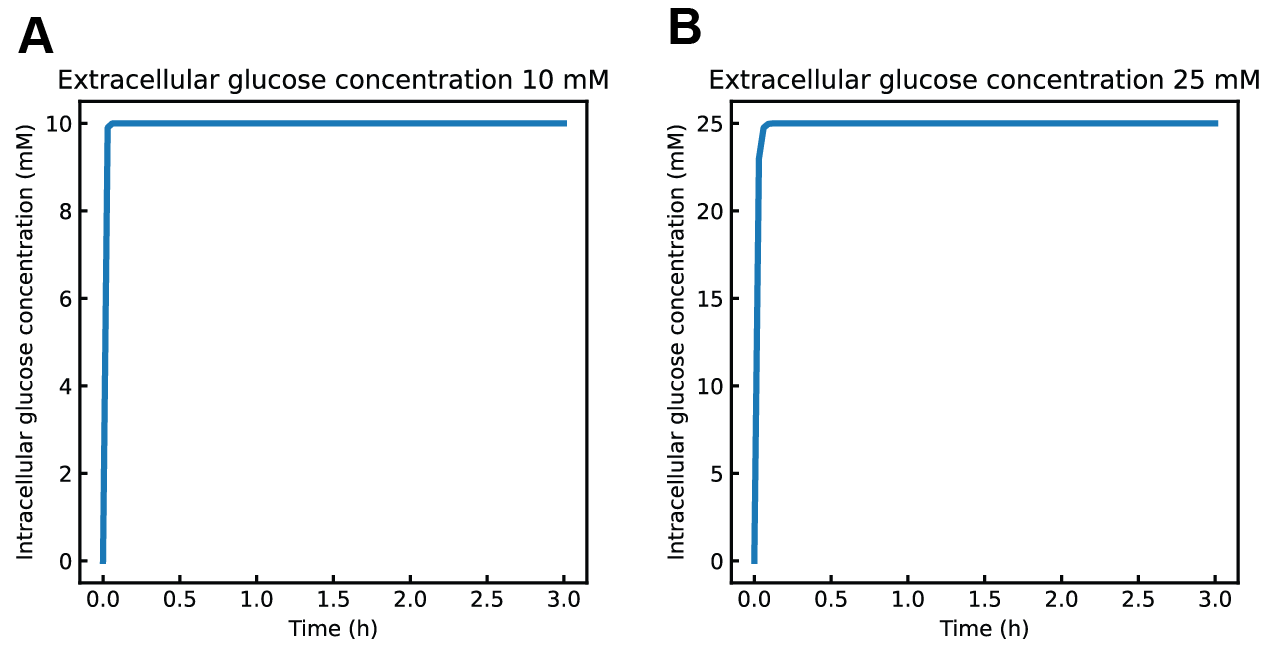


**Supplementary Text 2: Calculation of layers of glucose carryover on a cell surface**

To calculate how many layers of glucose molecules will stack on a single cell based on our estimated glucose carryover per cell surface area, we first estimated the theoretical size of the molecule of glucose. Typically, the glucose molecule could be seen as a hexagon ring with a carbon-carbon bond length *a* ≈ 0.15 nm (<http://book.bionumbers.org/how-big-are-biochemical-nuts-and-bolts/>). Then the area *A* of this hexagon is:

$$\begin{aligned} A= \frac{3\sqrt{3}}{2}a^{2}=0.06 {nm}^{2}\#\left( \mathrm{SI}.4 \right) \end{aligned}$$

With the average cell surface area of the *hxk*-null strain (used in the estimation of glucose carryover per surface area) *S* = 36.98 µm^2^, if glucose molecules are uniformly spread on the surface of a cell, the theoretical number of glucose molecules on a single layer is provided by:

$$\begin{aligned} N_{glc, per-layer}=\frac{S}{A}=6.33\times{10}^{8}\#\left( \mathrm{SI}.5 \right) \end{aligned}$$

We estimated a coefficient of average glucose carryover per cell surface area ($\frac{n_{glc,co}}{S}$)*_hxk-null_* = 0.87 µmol·m^-2^, together with the average cell surface area *S*, and Avogadro constant *N_A_*, we calculated the number of glucose carryover molecules per cell:

$$\begin{aligned} N_{glc,per-cell}=\left( \frac{n_{glc,co}}{S} \right)_{hxk-null}\cdot S\cdot N_{A}=1.94\times{10}^{10}\#\left( \mathrm{SI}.6 \right) \end{aligned}$$

Eventually, with the total number of glucose carryover molecules on a cell and the number of glucose carryover molecules per layer, we calculated layers *L* of glucose carryover on a single cell if all carried over glucose stacks on the cell surface:

$$\begin{aligned} L= \frac{N_{glc,per-cell}}{N_{glc,per-layer}}=30.63\#\left( \mathrm{SI}.7 \right) \end{aligned}$$
